# Expansion and diversity of caspases in *Mytilus coruscus* contribute to larval metamorphosis and environmental adaptation

**DOI:** 10.1186/s12864-024-10238-w

**Published:** 2024-03-27

**Authors:** Yanfei Cao, Linxiang Xu, Xinwei Xiong, Xiao Liu

**Affiliations:** National Engineering Research Center For Marine Aquaculture, Zhejiang Ocean University, Zhoushan, Zhejiang 316022 China

**Keywords:** Caspase, Apoptosis, Gene expansion, Tandem duplication, Functional differentiation, *Mytilus coruscus*

## Abstract

**Background:**

Apoptosis is involved (directly and indirectly) in several physiological processes including tissue remodeling during the development, the turnover of immune cells, and a defense against harmful stimuli. The disordered apoptotic process participates in the pathogenesis of various diseases, such as neoplasms, and chronic inflammatory or systemic autoimmune diseases, which are associated with its inadequate regulation. Caspases are vital components of the apoptotic pathway that are involved in developmental and immune processes. However, genome-wide identification and functional analysis of caspase have not been conducted in *Mytilus coruscus*, which is an economically important bivalve.

**Results:**

Here, 47 caspase genes were identified from the genomes of *M. coruscus*, and the expansion of caspase-2/9 and caspase-3/6/7 genes were observed. Tandem duplication acts as an essential driver of gene expansion. The expanded caspase genes were highly diverse in terms of sequence, domain structure, and spatiotemporal expression profiles, suggesting their functional differentiation. The high expression of the expanded caspase genes at the pediveliger larvae stage and the result of apoptosis location in the velum suggest that the apoptosis mediated by them plays a critical role in the metamorphosis of *M. coruscus* larvae. In gill, caspase genes respond differently to the challenge of different strains, and most caspase-2/9 and caspase-3/6/7 genes were induced by copper stress, whereas caspase-8/10 genes were suppressed. Additionally, most caspase genes were upregulated in the mantle under ocean acidification which could weaken the biomineralization capacity of the mantle tissue.

**Conclusions:**

These results provide a comprehensive overview of the evolution and function of the caspase family and enhanced the understanding of the biological function of caspases in *M. coruscus* larval development and response to biotic and abiotic challenges.

**Supplementary Information:**

The online version contains supplementary material available at 10.1186/s12864-024-10238-w.

## Background

Apoptosis, an evolutionally conserved programmed cell death pathway, serves as a homeostatic mechanism for maintaining cell populations or a defense mechanism against harmful stimuli [1]. Caspases, the main elements involved in apoptosis, are members of a family of cysteine proteases that manage intermediate and late stages of apoptosis [2]. Twelve confirmed caspase genes have been confirmed in humans [3] and 19 have been identified in zebrafish [4]. However, only seven and four caspase genes are retained in *Drosophila melanogaster* and *Caenorhabditis elegans* due to lineage-specific losses [5]. In mollusks, 40 caspase genes have been reported in the *Crassostrea gigas* [6], 30 have been identified in the *Chlamys farreri* [7], and 3 initiator and 4 executioner caspases also were characterized in the mussel *Mytilus galloprovincialis* [8, 9]. The structure and function show differences among the caspase family members. Based on the functional and structural features, mammalian caspases can be divided into four groups, namely, apoptosis initiators (caspase-2/8/9/10), apoptosis executioners (caspase-3/6/7), inflammatory caspase (caspase-1, 4, 5, 11, 12, and 13) [10], and keratinocyte differentiation-related caspase (caspase-14) [11]. They include an N-terminal prodomain of varying sizes and a conserved CASc domain with a large catalytic p20 and a small regulatory p10 subunit [5]. Initiator and inflammatory caspases contain a long prodomain with homotyphic interaction motifs, such as death-effector domain (DED) in caspase-8/10 and caspase-recruitment domain (CARD) in caspase-2/9 and inflammatory caspases [5]. The p20 subunit contains a pentapeptide active-site motif consisting of Gln–Ala–Cys–X–Gly (X is Arg, Gly, or Gln) [12, 13]. Apoptosis can be triggered by two major apoptotic pathways: an extrinsic pathway involving death domain-containing transmembrane receptors and the intrinsic pathway which implies the release of cytochrome c (Cyt c) from the mitochondria. Typically, caspases are synthesized in the cell as inactive zymogens that are activated following the cleavage event of specific aspartic acid residues and oligomerization. Activated initiator and executioner caspases gain proteolytic activity when they are cleaved at the linker that separates their large and small catalytic subunits. The activated caspase is a tetramer composed of two p20 and two p10 subunits, which contains the catalytic dyad Cys and His residues located in p20, whereas Arg and Gln, which tethers the carboxylate side chain of the essential Asp, are derived from both the p20 and p10 subunits [12].

Caspases drives cell death, including apoptosis and pyroptosis. The caspase-9 homologue in *D. melanogaster* regulates apoptosis, thereby controlling the dismantling of specific tissue and organogenesis during metamorphosis [14]. Caspase-1 triggers pyroptosis after activation by various inflammasomes and leads to the lysis of the affected cells [15]. A growing body of evidence suggests that apoptotic caspase possesses distinct non-apoptotic functions in addition to the core cell death function, including developmental processes [16], immune response [17], and cell proliferation versus differentiation [18, 19]. The inhibition of caspase activation during periods of neural vulnerability, both during apoptotic and non-apoptotic processes, is among the main targets in maintaining normal number, morphology, and function of neurons in the developing brain that have been exposed to environmental stress [20]. Caspases can serve as mechanisms to inhibit or reverse the harmful effects of injurious stimuli. For instance, caspase-8 can regulate inflammatory cytokine expression, inflammasome activation and cleavage of IL-1β, and protection against shock and microbial infection [21]. Caspase from both vertebrates and invertebrates can be activated by immune stimuli and functions as a recognition molecule to bind diverse pathogen associated molecular patterns [22–25]. Moreover, caspase-3 plays a key role in regulating the growth and homeostatic maintenance of normal and malignant cells and tissues in multicellular organisms [26].

Marine bivalve mollusks reside in highly dynamic environments and have limited mobility, are highly susceptible to pathogens, climate changes, and pollutants in the surrounding environment. Their remarkable adaptation to highly variable or stressful environments is not well understood at the molecular and genomic levels [27]. They possess a phenomenal capacity to control apoptotic caspase signal mechanisms for maintaining body homeostasis in response to environmental demands [28, 29]. The cell’s stress-sensing capacity determines the distinct cell fate, the cell adapts and survives, or the cell dies. More recently, the evolutionary role of caspases as essential stress sensors and survival factors were confirmed [30]. In mollusks, several caspases are involved in apoptotic processes associated with developmental processes [6, 31], responses to pathogen infection [23, 32, 33], and environmental stressors [32, 34, 35]. Systematic characterization and functional analysis of caspases have also been reported in other bivalves, such as oyster *C. gigas* [6] and scallop *C. farreri* [7], mussels *M. galloprovincialis* [8, 9] and *M. edulis* [36]. Interestingly, compared with vertebrates, caspase family genes are significantly expanded in oysters and scallops, which may help them adapt to complex living environments. However, less attention has been paid to the caspases in *M. coruscus*. The role of caspase signal mechanisms in cell death and non-death in *M. coruscus* is tremendously unknown.

*M. coruscus* is an economical artificial cultured bivalve in the East China Sea and is often treated as a potential candidate for environmental monitoring, ecotoxicological investigations, and chemical pollutant assessments. The caspases in *M. coruscus* were studied by characterizing the caspase family members *via* bioinformatics and phylogenetic analyses. In total, 47 caspase genes were identified, which underwent expansion driven by tandem duplication. The apoptosis occurrence in the larval velum was confirmed *via* TUNEL apoptosis detection. Moreover, the transcriptomic responses of caspases were illustrated when the *M. coruscus* suffered from bacterial, copper, and ocean acidification (OA) stress. Collectively, caspases from *M. coruscus* were identified, and their potential roles for development and environmental stress response were determined. Our observations revealed that the expansion and diversification of caspase genes in *M. coruscus* developed their functions in larvae development and response to environmental stress, thereby improving their adaption under dynamic environments.

## Results

### Expansion of caspases in *M. coruscus*

**Fig. 1:**
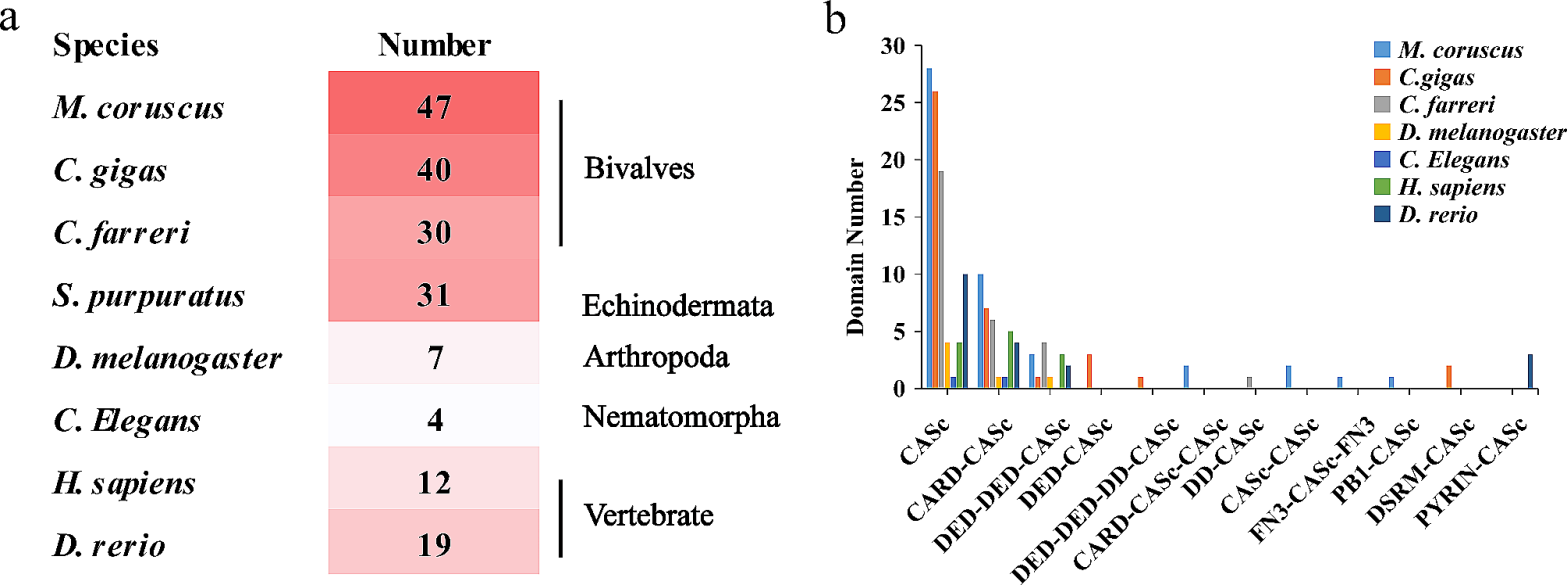
Expansion of caspase genes in bivalves. (**a**) Number of caspase genes in different species. (**b**) Domain combinations of caspases in different species. CASc, cysteine aspartase cysteine structural; CARD, caspase-recruitment domain; DED, death-effector domain; DD, death domain; DSRM, double stranded RNA-binding motif; FN3, Fibronectin type 3 domain; PB1, Phox and Bem1 domain

In total, 47 caspase genes were identified in *M. coruscus* (including two previously reported homologues) (Fig. 1a). This number is in line with the previous report, in which 40 and 30 caspase genes were identified in bivalves *C. gigas* and *C. farreri*. Compared with four model species (4–19 genes), an expansion of caspase genes was found in *M. coruscus*. SMART results showed that all the identified caspase genes from *M. coruscus* contained at least one typical CASc domain, 30 caspases did not have any specific domain within their prodomains, while 12 caspases contained CARD in their prodomains, and 3 caspases had two DED in their prodomains. (Fig. 1b). Interestingly, two caspases contained FN3 or PB1 domain in their prodomains, which are not found in the caspase of ecdysozoans and vertebrates and may support novel functions.

The expanded caspases from bivalves were highly diverse in domain structure. Seven different domain combinations were observed in the expanded caspase of *M. coruscus*, while all caspases from *Homo sapiens* and *Danio rerio* only contained three or four domain combinations (Fig. 1b). The deviations of domain combination types were associated with the duplication of the CASc domain, and four caspases contained two CASc domains. ScanProsite analysis revealed that all the 51 CASc domains of *M. coruscus* caspases contained at least one p20 or one p10 subunit. In comparison with ecdysozoans and vertebrates, the combination of p20 and p10 subunits of *M. coruscus* CASc domain was more diverse because of the deletion of p20 or p10 subunit and the addition of p20 subunit (Table S1). In *M. coruscus*, 26 CASc domains contained a large p20 and a downstream small p10 subunit, 19 CASc domains only contained p20, 4 CASc domains only contained p10 subunit, and 2 CASc domains contained two p20 and one p10 subunit (Table S1).

### Phylogenetic assessment of caspases in *M. coruscus*

**Fig. 2:**
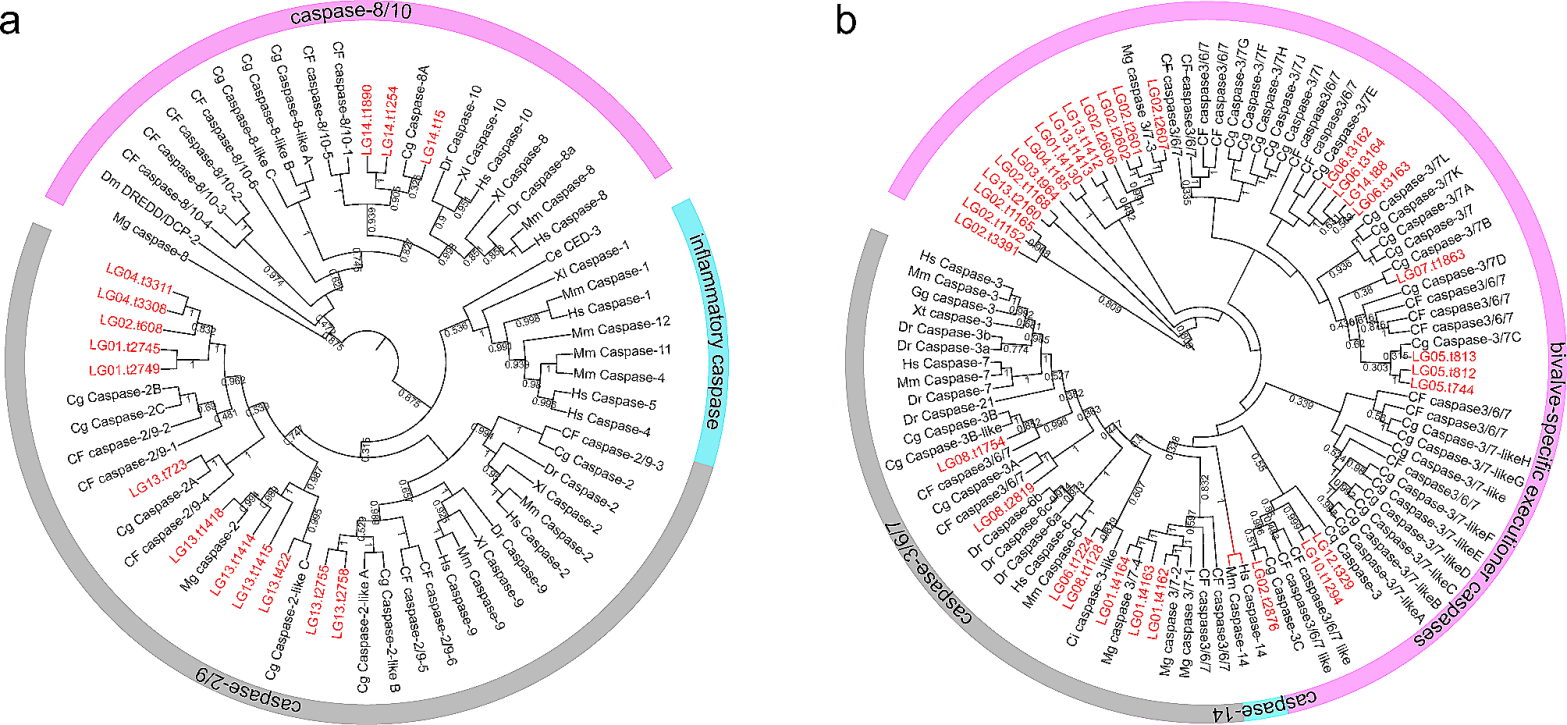
Phylogenetic trees of caspases from bivalves, vertebrate and other invertebrate. (**a**) Phylogenetic tree with initiator caspases and inflammatory caspases; (**b**) Phylogenetic tree with executioner caspases and caspase-14. Low support values (values less than 0.3) are not shown. The red font represents the caspase gene in *Mytilus coruscus*. LG, *Mytilus coruscus*; Cg, *Crassostrea gigas*; CF, *Chlamys farreri*; Mg, *Mytilus galloprovincialis*; Dm, *Drosophila melanogaster*; Ce, *Caenorhabditis elegans*; Ci, *Ciona intestinalis*; Hs, *Homo sapiens*; Dr, *Danio rerio*; Xl, *Xenopus tropicalis*; Mm, *Mus musculus*; Gg, *Gallus gallus*

Domains containing CARD or DED that possess *M. coruscus* caspase homologues were aligned with known initiator caspases-2, -9, -8, and − 10 of other species and vertebrate inflammatory caspases-1, -4, and − 5, which also contain CARDs. The remaining caspase homologues were aligned with known executioner caspase-3, -6, and − 7 and caspase-14 homologues. The phylogenetic analysis of initiator caspases included two clades, with one group including all CARD-containing caspases for caspase-2/9 and inflammatory caspases and a second group that included DED-containing caspase-8/10 homologues (Fig. 2a). In CARD-containing caspase clade, bivalve caspases clustered with vertebrate caspase-2/9, and no direct homologue was found in the inflammatory caspase group. In total, 18 bivalve caspase-2/9 individually clustered in a branch, 6 bivalve caspase-2/9 clustered with vertebrate caspase-9 in a branch, and 2 bivalve caspase-2/9 showed the highest homology to vertebrate caspase-2. In the caspase-8/10 clade, three caspase-8/10 genes in *M. coruscus* were perfectly clustered with previously reported caspase-8/10 (Fig. 2a). The phylogenetic analysis showed that the potential bivalve executioner caspases revealed distinct clustering of bivalve caspases, in which some clades are potentially unique to bivalves (Fig. 2b). None of the bivalve caspases have shown a direct homology to either of the vertebrate caspase-14. Altogether, 12 caspase-2/9 genes, 3 caspase-8/10 genes, and 32 executor caspase genes were identified in *M. coruscus*, and caspases-2/9 and caspases-3/6/7 have higher copy numbers than those in vertebrates (Table S2).

### Chromosomal distribution, tandem duplication and gene structure of the caspase genes

**Fig. 3:**
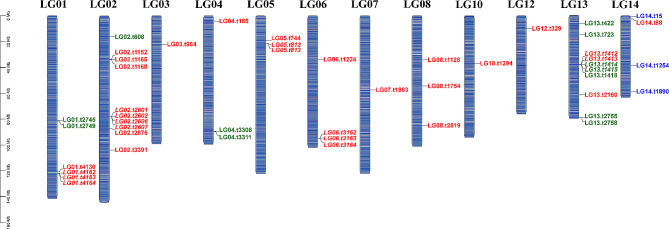
Chromosome distribution of the caspase family members in *Mytilus coruscus*. The red color indicates executioner caspases-3/6/7. The green color indicates initiator caspase-2/9. The blue color indicates initiator caspase-8/10. Italics represent tandem replicated genes

The caspase family members in *M. coruscus* were distributed across 12 of the 14 chromosomes (Fig. 3). Far from being uniformly split among chromosomes, the caspase genes displayed a highly skewed distribution, in which more than 50% of caspase genes were located on chromosomes LG01, LG02, and LG13 (Fig. 3). Chromosomes LG02 and LG13 contained 10 caspase genes each, and six genes were located in LG01. The other chromosomes encoded as few as 1–4 genes. The localization of caspase genes in the genome clearly showed that different types of caspase genes were not evenly distributed. The three initiator Mc-caspase-8/10 genes were distributed on chromosome LG14, while initiator Mc-caspase-2/9 genes were located on chromosomes LG01, LG02, LG04, and LG13. The executioner Mc-caspase-3/6/7 genes were distributed on 12 chromosomes, the number ranged from 1 to 9.

Among the 47 caspase genes in *M. coruscus*, 16 (34.04%) were linked in tandem arrays including five two-gene pairs, and two three-gene arrays (Table 1; Fig. 3). These tandem arrays were located on chromosomes LG01, LG02, LG05, LG06, and LG13 (Fig. 3). Among the tandem duplication genes, 14 genes were executioner Mc-caspase-3/6/7, and two genes were initiator Mc-caspase-2/9. Therefore, tandem duplication is a major contributor to the expansion of executioner caspase and caspase-2/9 genes. Furthermore, tandem repeat genes differed in spatiotemporal (Fig. S1), suggesting differences in regulatory elements and possibly function.

**Table 1:** Tandem duplication of caspase genes in *Mytilus coruscus*

Type	No. of Arrays	No. of Genes
2-gene array	5	10
3-gene array	2	6
Total	6	16 (34.04%)

Exon–intron structure analysis revealed that most caspase genes contain multiple introns (intron > 2) in *M. coruscus*. In total, 45 (95.74%) caspases were intron-rich gene, and one caspase (LG01.t4130) contained 2 introns. One caspase (LG02.t2606) was an intronless gene (Fig. S2), and LG02.t2606 and LG02.t2607 formed a two-gene tandem array (Table 1; Fig. 3).

### Sequence diversity of caspases in *M. coruscus*

**Fig. 4:**
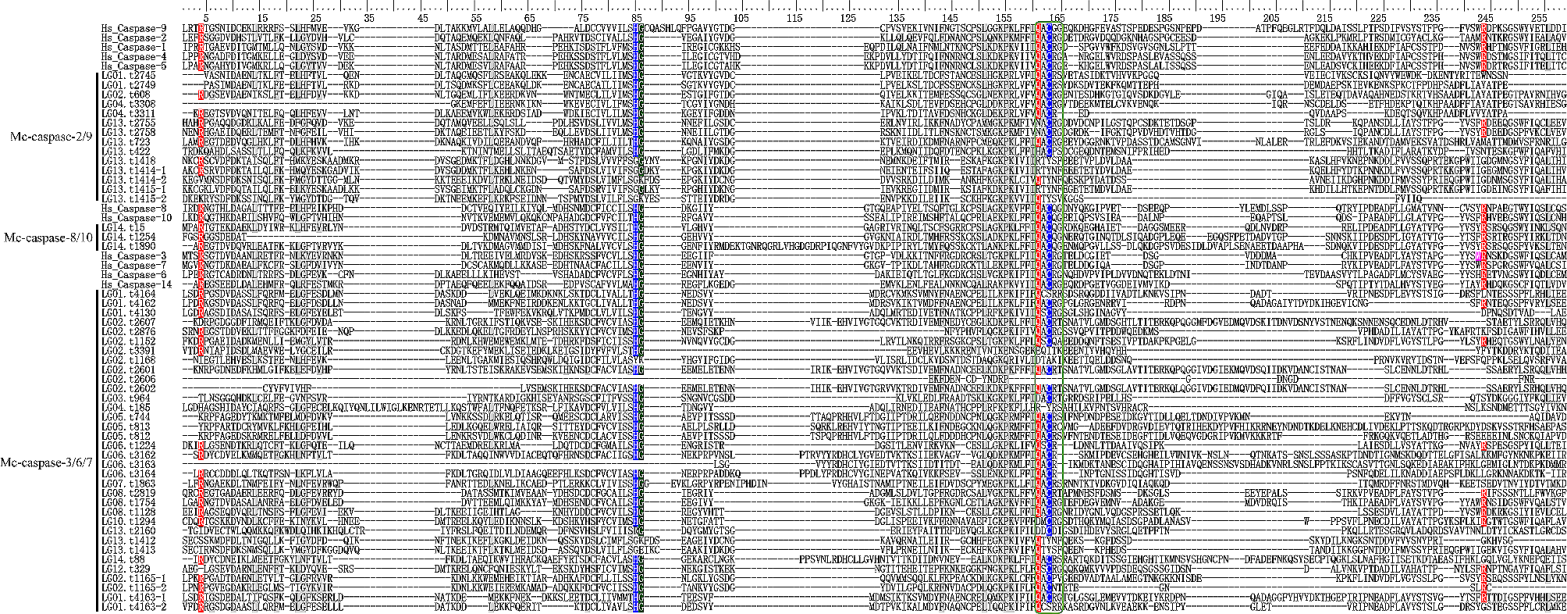
Multiple sequence alignment of caspases from *Mytilus coruscus* and *Homo sapiens*. The catalytic dyad (blue), residues that participate in the stabilization of the carboxylate side chain of the Asp (red), the pocket physical shape of the P4-binding site of caspase-3 is partially conferred by Trp (pink), and residues that contribute to the ‘oxyanion hole’ (green) are indicated. Green boxes represent pentapeptide sites

Fifteen initiator caspases and 32 executioner caspases were identified in *M. coruscus*. The expansion has resulted in the high sequence diversity of the expanded initiator Mc-caspase-2/9 and executioner Mc-caspase-3/6/7 genes (Fig. 4). Multiple sequence alignment of CASc domains showed that only 11 CASc domains were identical to human caspases CASc domain with pentapeptide sites Gln–Ala–Cys–X–Gly (X is Arg, Gly, or Gln) motif, and 40 (78.4%) CASc domains did not contain Gln, Ala, Cys, or Gly in the pentapeptide active-site. Three residues (Arg, Gln, and Arg) in all vertebrate caspases are tethered by hydrogen bond interactions with the carboxylate side chain of the Asp in the substrate. In *M. coruscus*, the Arg and Gln residues of Mc-caspases responsible for the interaction with Asp in the substrate were deleted or mutated in many Mc-caspases. Nine Mc-caspase-2/9, 3 Mc-caspase-8/10, and 26 Mc-caspase-3/6/7 were identical to human caspases with a catalytic dyad (His and Cys residue) (Table S1). The other Mc-caspases displayed very different catalytic dyad. His residues in three caspase-2/9 genes were mutated to Gly, and Cys was mutated to Thr. His or Cys residues in eight Mc-caspase-3/6/7 genes were deleted or mutated.

### Spatiotemporal expression of caspase in *M. coruscus*

**Fig. 5:**
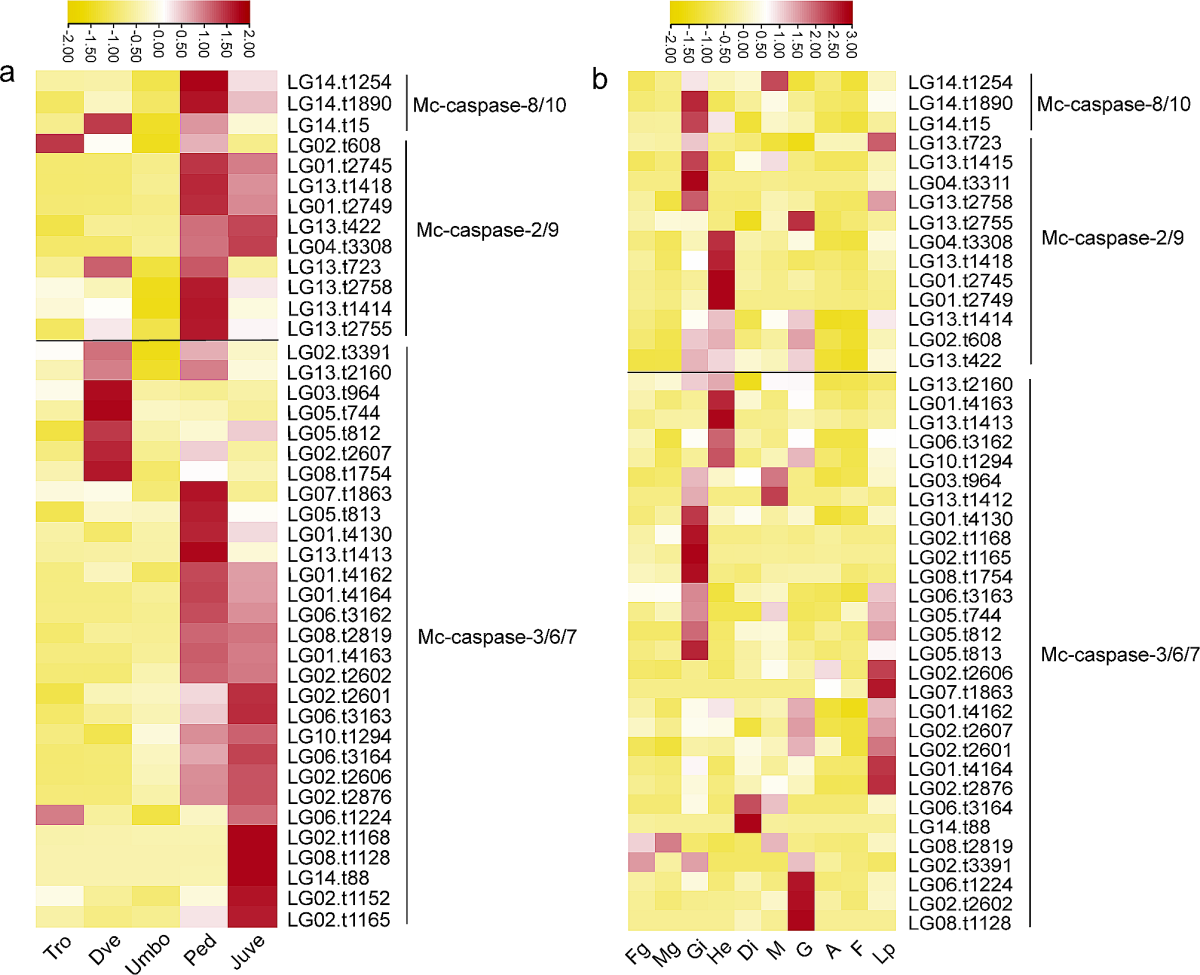
Diversity in the temporal and spatial expression of caspase genes in *Mytilus coruscus*. (**a**) Heat map caspase expression during development. (**b**) Expression patterns of caspase genes in different tissues. Tro, trochophore; Dve, D-larva; Umbo, umbo larva; Ped, pediveliger; Juve, juvenile; Fg, female gonad; Mg, male gonad; Gi, gill; He, hemocyte; Di, digestive gland; M, mantle; G, gut; A, adductor muscle; F, foot; Lp, labial palp

The expression patterns of Mc-caspases in different tissues and development larvae were systematically characterized by re-analyzing the published transcriptome of *M. coruscus*. Overall, the expression of Mc-caspase genes was tissue- and development stage-specific (Fig. 5, Table S4). A total of 41 caspase genes were detected at different development stages (FPKM > 1). One Mc-caspase-2/9 and one Mc-caspase-3/6/7 genes were highly expressed at the trochophore stage. Seven Mc-caspase-3/6/7 genes, one Mc-caspase-2/9 gene, and one Mc-caspase-8/10 gene were highly expressed at the D-larval stage. Two Mc-caspase-8/10 genes and six Mc-caspase-2/9 genes were highly expressed at the pediveliger stage. Most Mc-caspase-3/6/7 genes were highly expressed at the pediveliger and juvenile stages (Fig. 5a). Furthermore, strong apoptosis signals were distributed in the velum of the pediveliger larvae according to the result of apoptosis testing by using TUNEL method (Fig. S3).

For different tissues, two Mc-caspase-8/10 and three Mc-caspase-2/9 genes were specifically and highly expressed in the gill. Four Mc-caspase-2/9 genes were specifically and highly expressed in the hemocytes. Only one Mc-caspase-8/10 gene was highly expressed in the mantle, and one Mc-caspase-2/9 was highly expressed in the gut (Fig. 5b). Twelve Mc-caspase-3/6/7 genes were highly expressed in the gill, and five Mc-caspase-3/6/7 genes were highly expressed in the hemocytes along with some Mc-caspase-3/6/7 genes were highly and specifically expressed in other tissues (Fig. 5b).

### Expression of Mc-caspases under bacterial and environmental stress

**Fig. 6:**
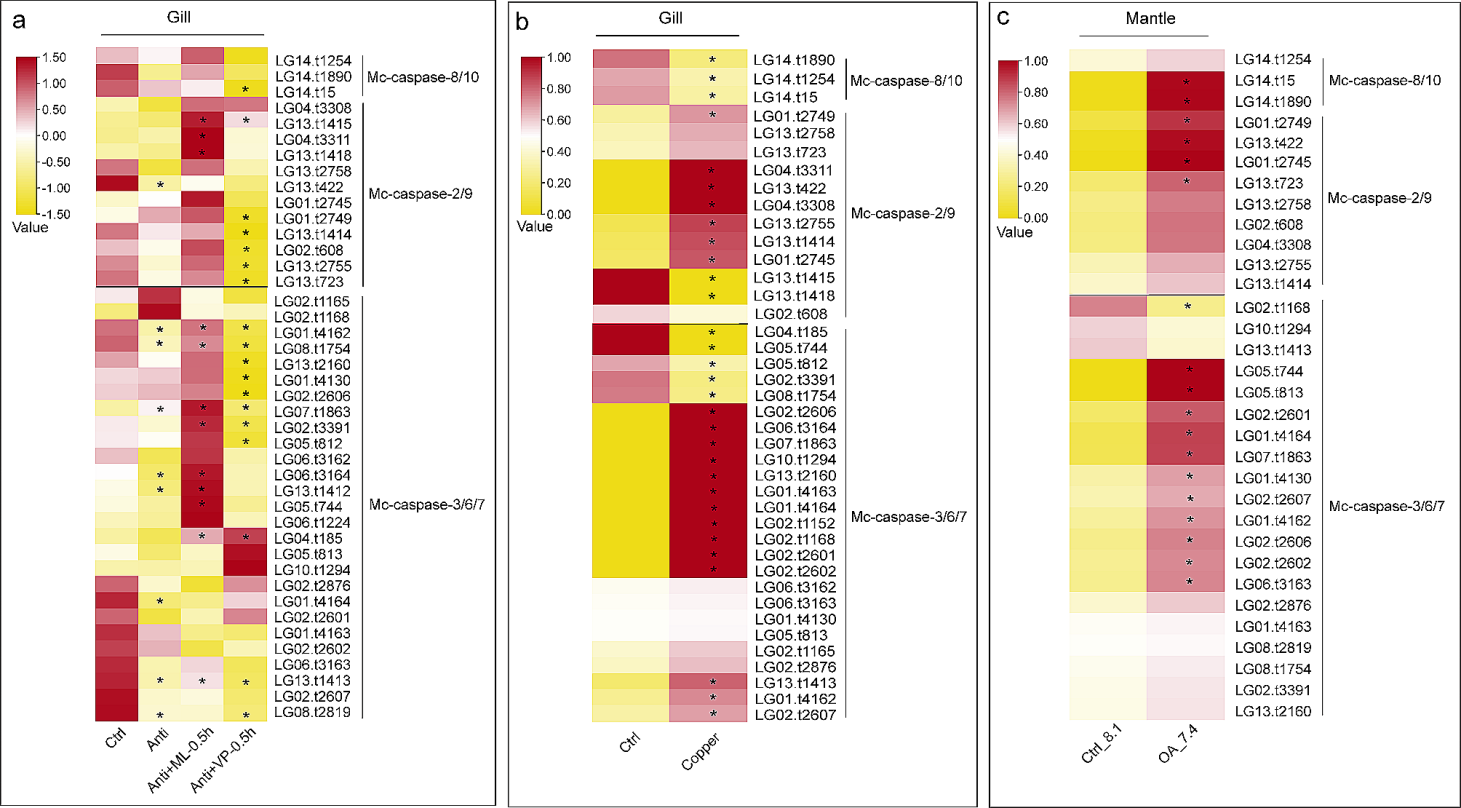
Expression profiles of caspase homologues in *Mytilus coruscus* after different stress. (**a**) Expression levels of the caspase genes in gill after the antibiotic and bacterial challenge. (**b**) Expression levels of the caspase genes in gill exposure to copper. (**c**) Expression levels of the caspase genes in mantle after ocean acidification treatment. Ctrl, control; Anti, exposure to a mixture of antibiotics for 24 h; ML, *Micrococcus luteus*; VP, *Vibrio parahemolyticus*; Anti + ML/VP-0.5 h, *M. luteus* or *V. parahemolyticus* challenge was performed 0.5 h after exposure to antibiotics for 24 h; OA, ocean acidification. * indicates significant difference at fold change ≥ 2

To explore the expression pattern of Mco-caspase genes under a mixture of antibiotics and bacterial challenges, we downloaded the transcriptome data from NCBI after mixed treatment with four antibiotics (ampicillin, kanamycin, gentamycin, and streptomycin) for 24 h, and *Micrococcus luteus* or *Vibrio parahemolyticus* challenge for 0.5 h after mixed treatment with four antibiotics for 24 h. In total, 42 Mc-caspase genes were detected in the gill before and after antibiotics and bacteria treatment (FPKM > 1). In comparison with the control group, one Mc-caspase-2/9 gene and seven Mc-caspase-3/6/7 genes were significantly downregulated after antibiotics treatment for 24 h (Fig. 6a, Fold change > 2). Compared with the antibiotic treatment group, most of the caspase genes were upregulated after *M. luteus* (Gram-positive bacteria) challenge and downregulated after *V. parahemolyticus* (Gram-negative bacteria) challenge (Fig. 6a). Three Mc-caspase-2/9 and 9 Mc-caspase-3/6/7 genes were significantly induced at 0.5 h after *M. luteus* challenge (Fig. 6a, Fold change > 2). One Mc-caspase-8/10, 5 Mc-caspase-2/9 genes, and 10 Mc-caspase-3/6/7 genes were significantly decreased at 0.5 h after *V. parahemolyticus* challenge (Fig. 6a, Fold change > 2). Therefore, the Mc-caspase genes responded differently to the stimulation of different bacteria in the gill.

The expression of caspase genes in gill under copper (0.2 ppm) challenge was also analyzed. Three Mc-caspase-8/10 genes were significantly downregulated, while seven Mc-caspase-2/9 genes were significantly upregulated in gill after exposure to copper (Fig. 6b, Fold change > 2). Fourteen Mc-caspase-3/6/7 genes were significantly upregulated, and 5 Mc-caspase-3/6/7 genes were significantly downregulated after exposure to copper. Therefore, different types of caspases have different responses to the copper challenge.

The expression of caspase genes in the mantle after mussels were exposed to the ambient pH (8.1) or a lowered pH (7.4) for 40 days was also compared. OA dramatically affects the expression of Mc-caspase genes in the mantle. A total of 32 Mc-caspase genes were detected in the mantle after OA treatment (FPKM > 1). All initiator Mc-caspase and most executioner Mc-caspase genes were induced after OA treatment (Fig. 6c). Among them, two Mc-caspase-8/10, four Mc-caspase-2/9 genes, and 11 Mc-caspase-3/6/7 genes were significantly upregulated by 2.8-4.0 folds compared with those in the control group (Fig. 6c, Fold change > 2).

## Discussion

Caspase family members are involved in apoptosis, cell differentiation and proliferation, inflammation, development, and immune homeostasis. This study represents a comprehensive set of genomic and transcriptomic data that revealed the expansion and diversity of caspase genes in *M. coruscus* compared with ecdysozoans and vertebrates. In total, 47 caspase genes were identified from the genome of *M. coruscus*. This number is in line with the previous report among 40 caspase genes in *C. gigas* [6], and 30 genes in *C. farreri* [7]. An expansion event of caspase genes was observed in the genome of *M. coruscus*, which was consistent with that observed in other bivalves [6, 7]. The mammalian caspases are subclassified into four types, namely, initiators, executioners, inflammatory caspase, and keratinocyte differentiation-related caspase. However, based on the structural versus sequential characteristics and the phylogenetic tree analysis, only 15 initiators caspase genes and 32 executioner caspase genes were identified in *M. coruscus*, and inflammatory and keratinocyte differentiation-related caspases were not observed. Our analysis also indicated that tandem duplication contributed to the expansion of these caspase genes in *M. coruscus*, as indicated by tandem arrays. Expanded caspases in oysters originate from gene duplication [6]. Bivalves reside in highly dynamic environments and require a remarkable capacity to maintain body homeostasis in response to environmental demands. A recent study has proposed that the complex regulation of apoptosis reflects dynamic competition for control between the host and pathogens [27]. The presence of multiple initiator and executioner caspases may reflect the bivalve-specific expansion of these proteins, highlighting their importance in bivalves’ adaptation to stationary life under variable environments.

The expansion of caspase genes in *M. coruscus* through tandem duplication has led to extensive diversity in domain structure and sequence. Some *M. coruscus* caspases lose the p10 or p20 subunit. Vogeler et al. also found some p10-minus caspases in oysters [6]. The p20-minus caspase-2 was also found in Mytilus galloprovincialis [32]. These genes may experience p20 or p10 subunit loss during evolution. Structures similar to p10-minus caspase have been previously reported in non-metazoan species as metacaspase-like proteins, in which metacaspases are caspase homologues that are present in prokaryotes to higher plants [37, 38]. The metacaspase-like proteins of non-metazoan are suggested to be non-functional or potentially vary in their substrate specificity to traditional caspases [38]. Further research is needed to clarify whether these unusual *M. coruscus* caspases are functional specifically. Caspases recognize a very short tetrapeptide sequence (P4-P3-P2-P1) in target substrate polypeptides. For example, caspases-3 and − 7 share preferences for substrate sequences that contain DXXD (X is any amino acid) [39]. In almost all caspase substrates, P1 is clearly Asp but rarely Glu, and Asp is anchored by hydrogen bonds *via* Arg and Gln residues located in the p20 and p10 subunits [39, 40]. The P2 and P3 binding sites are fairly unique but tolerate a wide range of substitutions. The P4 binding site within the p10 subunit is a main determinant of substrate specificity that varies significantly among different caspase family members. This property is best exemplified in the comparison between caspase-1 and caspase-3. The P4 binding site of caspase-1 is a large shallow depression on the surface of the protease that easily accommodates a large number of hydrophobic residues, while the P4 binding site of caspase-3 has a well-defined and narrow pocket structure, and the physical shape of the pocket is conferred in part by Trp [40]. In *M. coruscus*, the Arg and Gln residues responsible for the interaction with Asp in the substrate were deleted or mutated in many caspases, suggesting that the amplified caspases may have broad substrate recognition properties. After substrate binding, the catalysis of the caspase is performed by a catalytic dyad consisting of Cys and His in p20 subunit [40]. The catalytic sites of some caspase-2/9 and caspase-3/6/7 in *M. coruscus* were mutated, suggesting that these genes may have unusual functions. Similar mutations were also found in *C. gigas* [6], *C. farreri* [7], and *M. galloprovincialis* [32], indicating that this variation is ubiquitous in bivalve caspases.

Sequence and domain structure diversity are essential for functional diversification. The functions of the caspase genes were further determined by analyzing the expression profiles of caspase genes in different larvae development and tissues. The expression of Mc-caspase genes was development stage and tissue-specific. Most initiator caspase and many executioner caspase genes were highly expressed at the pediveliger stage, and some genes were highly expressed at trochophore and D-larval stage. This finding indicates that the functional differentiation of the *M. coruscu*s caspases occurred, and the expanded caspase may be an important condition for the metamorphosis of the *M. coruscus* larvae. Caspases are particularly important during development as transducers and executioners of programmed cell death, because the elimination of unnecessary cells contributes to tissue morphogenesis [16]. Larvae metamorphosis involves a physiological versus irreversible transformation process from a planktonic larva to a sessile adult, which includes the degeneration of many larval tissues such as velum and the concurrent proliferation and differentiation of adult tissues such as gills. In the present study, strong apoptosis signal was observed in the cells of velum of the pediveliger larvae of *M. coruscus*. The degeneration of larval tissues is linked to caspase-dependent apoptosis [31, 41–43]. For example, in *C. angulata*, caspase-3 is located in the velum and foot tissues that will degenerate with growth of larvae [31]. Adrenaline is a potent inducer of metamorphosis in bivalve larvae. The expression of one Mc-caspase-3/6/7 gene in *M. coruscus* was upregulated in the pediveliger larvae after the incubation of adrenaline, and the induction of epinephrine-induced metamorphosis in pediveligers larvae was significantly reduced after this gene was knocked down [44]. Therefore, apoptosis in the larval velum may be mediated by caspases. The expression patterns of caspase genes in different tissues also support the functional differentiation of caspase genes. Overall, some caspase genes were specifically and highly expressed in the gill or hemocytes, while the other genes were specifically expressed in other tissues. Bivalves gill is the main interface between the organism and environment [45], and the hemocytes play a central role in the recognition of foreign agents and defense against bacterial invasion [46]. Thus, the exceptionally high expression of caspase genes in the *M. coruscus* gill and hemocytes may be an adaptation for rapid response to dynamic environmental conditions.

Bivalves lack adaptive immunity but thrive in microbe-rich environments as filter feeders, illustrating their remarkable tolerance to biotic and abiotic stresses [27]. The expansion and diversity of many immune and stress-related gene families may be central to adaption of bivalves to highly stressful and widely challenging environments [27, 47–52]. Our analysis showed that the expansion and high diversity of caspases may endow *M. coruscus* with an enhanced capacity to handle and respond to pathogen and environmental stimuli. *M. coruscus* is particularly resistant to bacterial infections and shows a remarkable specificity of the immune response toward different bacteria [53]. Most caspase genes including Mc-caspase-3/6/7 were significantly induced in the gill after *M. luteus* (Gram-positive strains) challenge but were suppressed after *V. parahemolyticus* (Gram-negative strains) challenge. These results suggesting that *M. luteus* activates gill cell apoptosis, while *V. parahemolyticus* suppresses gill cell apoptosis. Gram-negative strains are dominant in seawater, and *M. coruscus* has more sensitivity to Gram-positive bacteria than Gram-negative bacteria [53]. In *C. gigs*, caspase-3 is an LPS-specific intracellular immune receptor, and the interaction between caspase-3 and LPS specifically inhibits the apoptosis induced by caspase-3 [23]. LPS is the component of the cell membrane of Gram-negative strains. LPS in the cell membrane of *V. parahemolyticus* may interact with the expanded Mc-caspase-3, thereby inhibiting the apoptosis. The complex regulation of apoptosis reflects dynamic competition for control between the host and pathogens [27]. Apoptosis is part of the cellular immune response that eliminates the replicative niche of intracellular pathogens and resolves infections [54]. To disrupt apoptosis, pathogens have evolved multiple mechanisms [54]. The downregulation of caspase genes and the inhibition of apoptosis caused by Gram-negative strains may be one of the main reasons for the strong tolerance of *M. coruscus* to them. Apoptosis is an important and multifunctional process that is often considered a signal for pollution monitoring. Cu^2+^ is a major toxic contaminant in the marine environment, and apoptosis pathway plays a role in copper stress in *M. coruscus* [55]. In the present study, a large number of Mc-caspase-2/9 and Mc-caspase-3/6/7 genes were upregulated in the gill after the copper challenge, suggesting they may be involved in copper stress-induced apoptosis. Moreover, OA greatly affects the physiological functions of *M. coruscus* [56]. Mc-caspase genes were induced in mantle under OA, indicating that expanded caspases are involved in the OA-induced apoptosis of mantle cells. This result corresponds to the impaired shell-fabrication ability of mussels under OA environments [56].

## Conclusion

In conclusion, 47 caspase genes were identified from the genome of *M. coruscus*, and the expansion of caspase-2/9 and caspase-3/6/7 genes was observed. The expansion through tandem duplication has created remarkable diversity in terms of domain structure, sequence, and expression profile, which may support their diversified functions. The high expression of the expanded caspase genes in pediveliger larvae and the location of apoptosis signal in the velum of pediveliger larvae revealed their essential role in the larval metamorphosis of *M. coruscus*. Transcriptome analysis revealed the functional importance of expanded caspase genes in response to various biotic and abiotic environmental factors. Our work lays the foundation for further functional exploration of particular caspases in *M. coruscus*. Further study of these diverse caspase genes may identify their novel functions in larval development and environmental changes and improve our understanding of apoptotic signaling pathways.

## Methods

### Identification of caspase in *M. coruscus*

The genome assemblies and annotation files of *M. coruscus* were downloaded from the GigaDB [57]. Based on the conserved caspase domain (PF00656, Peptidase_C14), the complete set of the caspase family members in *M. coruscus* were identified using the HMMER program under the default parameters [58]. For annotation, all proteins were queried against the KEGG and Nr databases by using the basic local alignment search tool (BLAST, *E* value ≤ 1-e5), and the results with best scores for each query protein were accepted. InterProScan was used to predict the functional domain (Table S5) [59]. The genes annotated as caspase from *C. gigas* and *C. farreri*, *D. melanogaster*, *C. elegans*, *H. sapiens*, *D. rerio*, and *Strongylocentrotus purpuratus* were extracted from the National Center for Biotechnology Information (NCBI) database annotated as caspase genes. Caspase-specific domains were re-analyzed using the Simple Modular Architecture Research Tool (SMART) version 5.1 (http://smart.embl-heidelberg.de/smart/set_mode.cgi?NORMAL=1) and ScanProsite (https://prosite.expasy.org/scanprosite/). Only genes homologous with caspase (E-value ≤ 1-e5) and containing the CASc domain with p20 or p10 subunit were considered as caspases.

### Sequence and phylogenetic analysis

Multiple alignments of caspase protein sequences were conducted using the ClustalW program (http://www.genome.jp/tools-bin/clustalw). For phylogenetic tree construction, two main trees were constructed for the proposed initiator and executioner caspases based on the presence of CARD or DED containing prodomains. The MEGA 6.0 program was used to predict the best model for the generation of the phylogenetic tree. The Maximum-likelihood (ML) and Neighbor-joining (NJ) methods were used to construct phylogenetic trees for initiator and executioner caspases, respectively. Confidence values were obtained with bootstrapping with 1000 replications.

### Chromosome distribution, tandem duplication and gene structure analysis of caspase genes

Structural information for *M. coruscus* caspase genes was obtained from published data [57]. TBtools (v1.120) software was used to analyze the chromosome distribution of the caspase genes. The 3’ or 5’ of the tandemly duplicated caspases should contain at least one paralogous caspase genes. The exon and intron structure of caspase genes were determined using the Gene Structure Display Server (http://gsds.gao-lab.org/).

### Expression profile of caspase genes

The transcriptome data of developmental stages and different tissues, [57], the samples under antibiotics, bacteria [53], copper [55], and OA stress [56] were downloaded from the NCBI database (PRJNA689255, PRJNA578350, PRJNA707226, PRJNA301064, and PRJNA543748) and then re-analyzed (Table S3). Raw data were filtered using the Trimmomatic software to generate clean reads [60]. The clean reads were first mapped to the genomes of *M. coruscus* [57] by using the HISAT2 software [61]. Then, the sorted alignments were assembled and quantified *via* StringTie [62]. The gene expression levels of the caspase genes in *M. coruscus* were extracted from the profiles that were normalized to fragments per kilobase per million (FPKM). The heatmaps of gene expression were generated using Tbtools.

### Apoptosis detection in pediveliger larva

*M. coruscus* larvae were cultured at Ningbo Yinzhou Sanwan Aquatic Seed and Seedling Co., Ltd., and reared at 18 ℃. Paraformaldehyde solution (4%) was used to preserve pediveliger larvae (40 days) that were used for apoptosis detection experiments. TUNEL apoptosis detection Kit (Shanghai Yeasen BioTechnologies co., Ltd, Shanghai, China) was used to detect apoptosis in pediveliger larva. The apoptosis detection experiment was carried out according to the protocol. Fluorescence microscope (OLYMPUS BX53) was used to observe and photograph the distribution of apoptotic cells in pediveliger larvae.

### Electronic supplementary material

Below is the link to the electronic supplementary material.

Supplementary Material 1

Supplementary Material 2

Supplementary Material 3

Supplementary Material 4

## Data Availability

All transcriptome sequencing reads are openly available in NCBI SRA at https://www.ncbi.nlm.nih.gov/, reference number PRJNA689255, PRJNA578350, PRJNA707226, PRJNA301064, and PRJNA543748. The datasets used or analysed during the current study are mostly available in the Additional files.

## Abbreviations

CASc: Cysteine aspartase cysteine structural
CARD: Caspase-recruitment domain
DED: Death-effector domain
DD: Death domain
DSRM: Double stranded RNA-binding motif
FN3: Fibronectin type 3 domain
PB1: Phox and Bem1 domain
Tro: Trochophore
Dve: D-larva
Umbo: Umbo larva
Ped: Pediveliger
Juve: Juvenile
Fg: Female gonad
Mg: Male gonad
Gi: Gill
He: Hemocyte
Di: Digestive gland
M: Mantle
G: Gut
A: Adductor muscle
F: Foot
Lp: Labial palp
Ctrl: Control
Anti: Antibiotics
FPKM: Fragments Per Kilobase Million
OA: Ocean acidification
LG: *Mytilus coruscus*
Cg: *Crassostrea gigas*
CF: *Chlamys farreri*
Mg: *Mytilus galloprovincialis*
Dm: *Drosophila melanogaster*
Ce: *Caenorhabditis elegans*
Ci: *Ciona intestinalis*
Hs: *Homo sapiens*
Dr: *Danio rerio*
Xl: *Xenopus tropicalis*
Mm: *Mus musculus*
Gg: *Gallus gallus*
