# Supplementary material for: Expansion and diversity of caspases in Mytilus coruscus contribute to larval metamorphosis and environmental adaptation
Source: BMC Genomics. 2024 Mar 27;25:314. doi: 10.1186/s12864-024-10238-w (PMC10967218; doi:10.1186/s12864-024-10238-w)
Supplement: Supplementary file 1 — Supplementary Material 1 [file 12864_2024_10238_MOESM1_ESM.docx]

**Supplementary Information**

**Title: Expansion and diversity of caspases in *Mytilus coruscus* contribute to larval metamorphosis and environmental adaptation**

**Yanfei Cao, Linxiang Xu, Xinwei Xiong, Xiao Liu^*^**

National Engineering Research Center For Marine Aquaculture, Zhejiang Ocean University, Zhoushan, Zhejiang, 316004, China

^*^ Corresponding author.

Email address: [liuxiao@zjou.edu.cn](mailto:liuxiao@zjou.edu.cn) (X. Liu)


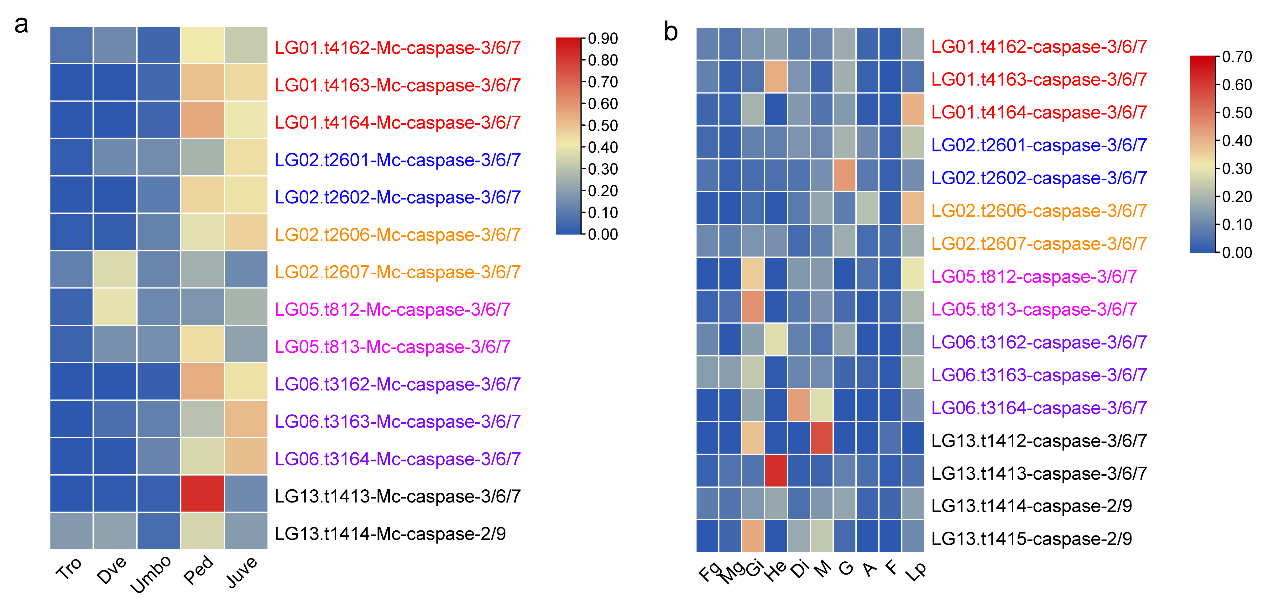


**Fig.S1.** Temporal and spatial expression of the tandem duplication of caspase genes in *M. coruscus*. a. Heat maps of expression at different developmental stages. b. Heat maps of expression in different tissues. Tro, trochophore; Dve, D-larva; Umbo, umbo larva; Ped, pediveliger; Juve, juvenile; Fg, female gonad; Mg, male gonad; Gi, gill; He, hemocyte; Di, digestive gland; M, mantle; G, gut; A, adductor muscle; F, foot; Lp, labial palp.


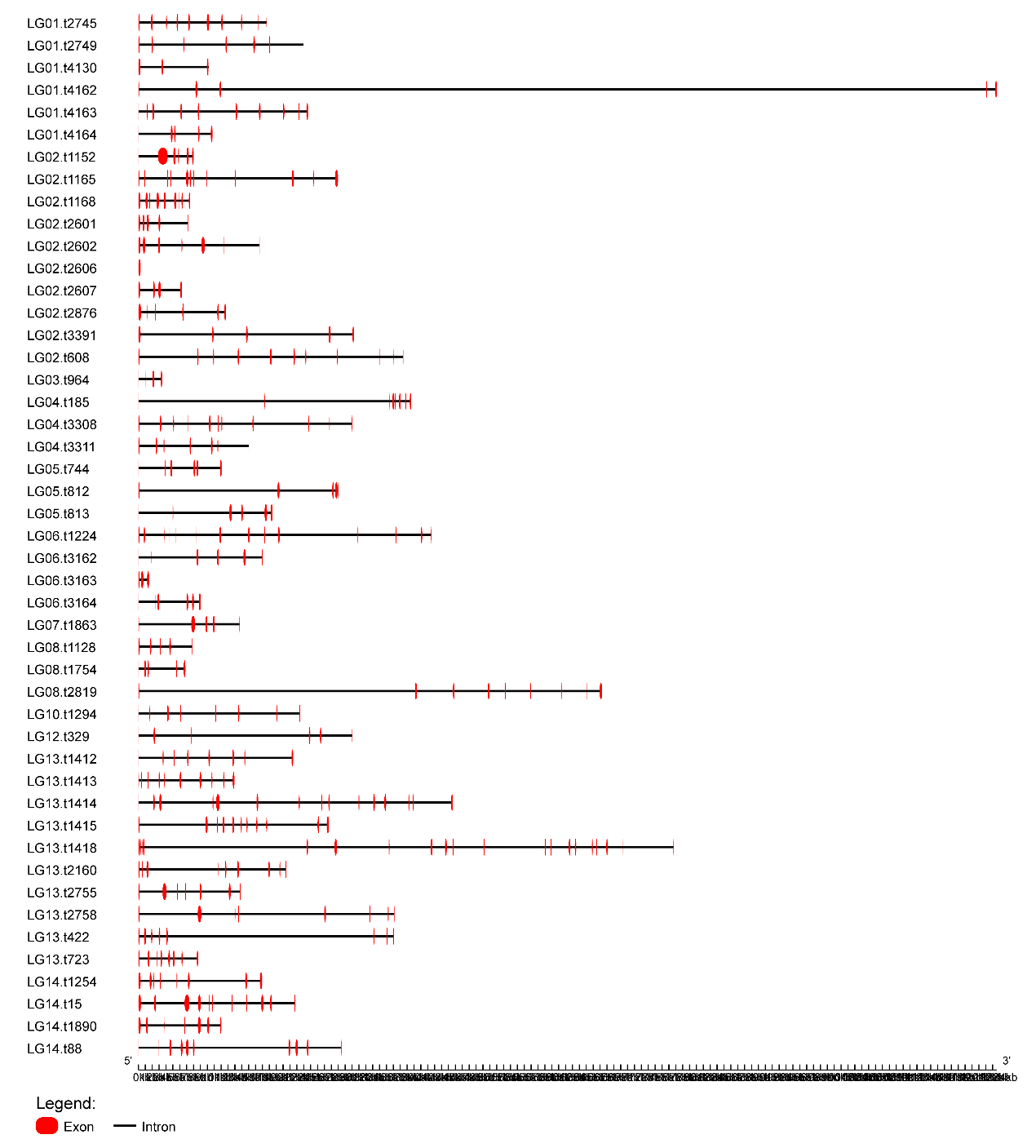


**Fig.S2.** Gene structure of caspase genes in *M. coruscus*.


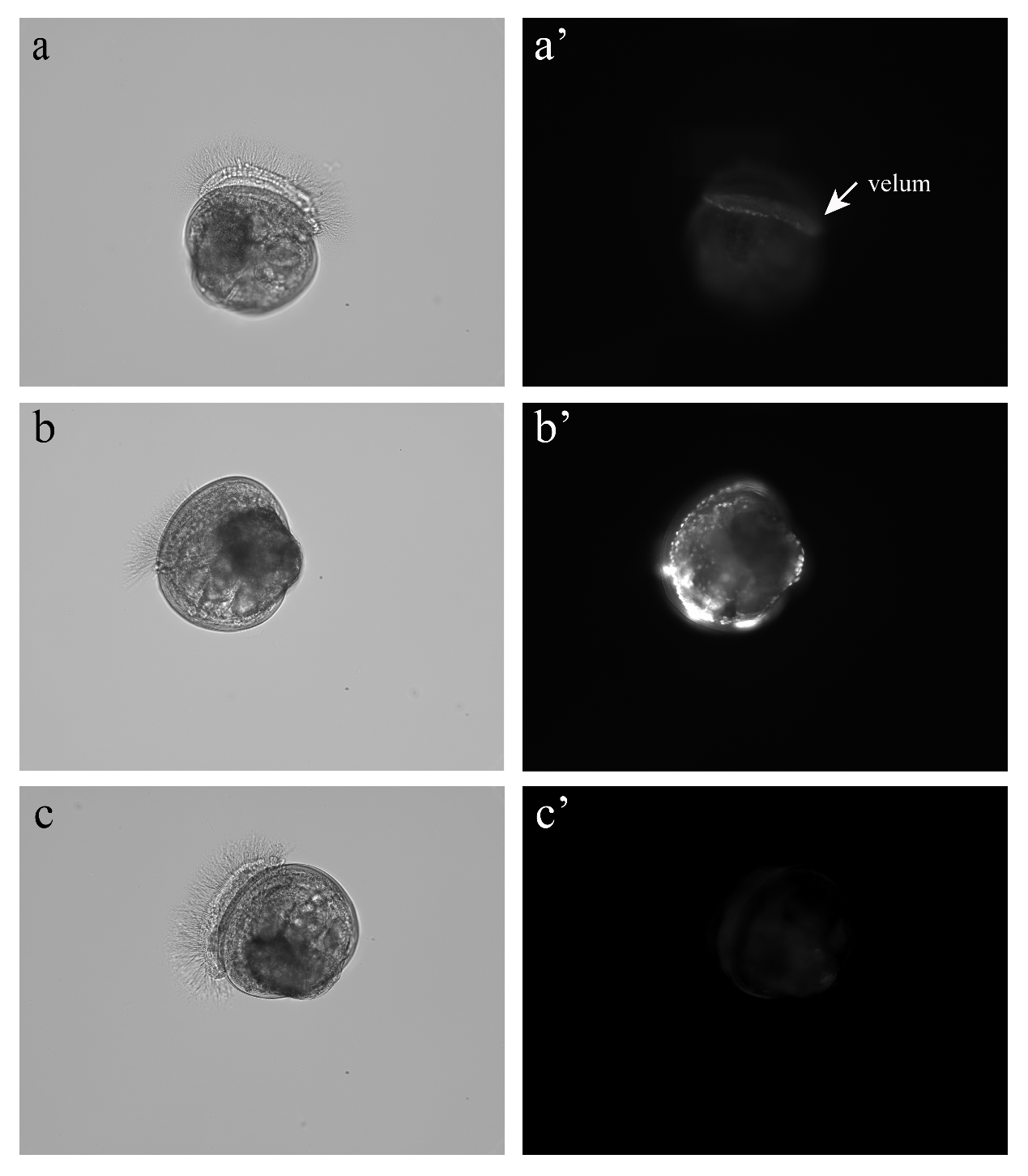


**Fig.S3.** The detection of apoptosis in pediveliger larva. a–a’, experimental group; b–b’, positive control group; and c–c’, negative control group.

**Table S1.** ScanProsite analysis revealed that all the 51 CASc domains of *M. coruscus* caspases.

| gene ID | Type | ScanProsite |
| --- | --- | --- |
| LG01.t2745 | caspase-2/9 | p20 |
| LG01.t2749 | caspase-2/9 | p20 |
| LG01.t4130 | caspases-3/6/7 | p20 |
| LG01.t4162 | caspases-3/6/7 | p20-p10 |
| LG01.t4163-1 | caspases-3/6/7 | p20-p10 |
| LG01.t4163-2* |  | p20-p10 |
| LG01.t4164* | caspases-3/6/7 | p20-p10 |
| LG02.t1152 | caspases-3/6/7 | p20-p10 |
| LG02.t1165-1 | caspases-3/6/7 | p20-p10 |
| LG02.t1165-2 | caspases-3/6/7 | p20 |
| LG02.t1168* | caspases-3/6/7 | p20 |
| LG02.t2601 | caspases-3/6/7 | p20-p10 |
| LG02.t2602 | caspases-3/6/7 | p20-p10 |
| LG02.t2606* | caspases-3/6/7 | p20 |
| LG02.t2607 | caspases-3/6/7 | p20-p10 |
| LG02.t2876 | caspases-3/6/7 | p20-p20-p10 |
| LG02.t3391* | caspases-3/6/7 | p20 |
| LG02.t608 | caspase-2/9 | p20-p10 |
| LG03.t964 | caspases-3/6/7 | p20 |
| LG04.t185* | caspases-3/6/7 | p20 |
| LG04.t3308 | caspase-2/9 | p20-p10 |
| LG04.t3311 | caspase-2/9 | p20 |
| LG05.t744 | caspases-3/6/7 | p20 |
| LG05.t812 | caspases-3/6/7 | p20 |
| LG05.t813 | caspases-3/6/7 | p20-p10 |
| LG06.t1224 | caspases-3/6/7 | p20-p10 |
| LG06.t3162 | caspases-3/6/7 | p20 |
| LG06.t3163* | caspases-3/6/7 | p20 |
| LG06.t3164 | caspases-3/6/7 | p20 |
| LG07.t1863 | caspases-3/6/7 | p20 |
| LG08.t1128 | caspases-3/6/7 | p20-p10 |
| LG08.t1754 | caspases-3/6/7 | p20-p10 |
| LG08.t2819 | caspases-3/6/7 | p20-p10 |
| LG10.t1294 | caspases-3/6/7 | p20-p10 |
| LG12.t329 | caspases-3/6/7 | p20-p10 |
| LG13.t1412* | caspases-3/6/7 | p20 |
| LG13.t1413* | caspases-3/6/7 | p20-p10 |
| LG13.t1418* | caspase-2/9 | p10 |
| LG13.t1414-1* | caspase-2/9 | p10 |
| LG13.t1414-2* |  | p10 |
| LG13.t1415-1* | caspase-2/9 | p10 |
| LG13.t1415-2* |  | p20 |
| LG13.t2160* | caspases-3/6/7 | p20-p10 |
| LG13.t2755 | caspase-2/9 | p20-p10 |
| LG13.t2758 | caspase-2/9 | p20-p10 |
| LG13.t422 | caspase-2/9 | p20-p10 |
| LG13.t723 | caspase-2/9 | p20-p10 |
| LG14.t1254 | caspase-8/10 | p20-p20-p10 |
| LG14.t15 | caspase-8/10 | p20-p10 |
| LG14.t1890 | caspase-8/10 | p20-p10 |
| LG14.t88 | caspases-3/6/7 | p20 |

Note: *represents caspase genes with mutations or deletions of amino acids on the catalytic dyad.

**Table S2.** Distribution of caspase gene homologues in different species. The numbers of expansion caspases in *M. coruscus* are highlighted in bold.

| **Gene Category** | **Gene Name** | ***H. sapiens*** | ***D. rerio*** | ***C. gigas*** | ***C. farreri*** | ***M. coruscus*** |
| --- | --- | --- | --- | --- | --- | --- |
| Inflammatory caspases | caspase-1/4/5/11/12/13 | 3 | 4 | 0 | 0 | 0 |
| Initiator caspases | caspase-2/9 | 2 | 4 | 7 | 6 | **12** |
|  | caspase-8/10 | 2 | 3 | 5 | 6 | 3 |
| Executioner caspases | caspase-3/6/7 | 3 | 7 | 28 | 18 | **32** |
| Keratinisation-related caspases | caspase-14 | 1 | 0 | 0 | 0 | 0 |
| other caspase | caspase-17 | 0 | 1 | 0 | 0 | 0 |
| Total number |  | 12 | 19 | 40 | 30 | 47 |
