# Supplementary material for: Expansion and diversity of caspases in Mytilus coruscus contribute to larval metamorphosis and environmental adaptation
Source: BMC Genomics. 2024 Mar 27;25:314. doi: 10.1186/s12864-024-10238-w (PMC10967218; doi:10.1186/s12864-024-10238-w)
Supplement: Supplementary file 2 — Supplementary Material 2 [file 12864_2024_10238_MOESM2_ESM.docx]

**Table S3** Summary of the data that collected from NCBI in this study.

| **Library Source** | **Accession number** | **Description** |
| --- | --- | --- |
| Developmental stages | SRR13364367 | Umbo larva 6 |
| Developmental stages | SRR13364368 | Umbo larva 1 |
| Developmental stages | SRR13364369 | D-larva 9 |
| Developmental stages | SRR13364370 | D-larva 8 |
| Developmental stages | SRR13364371 | D-larva 7 |
| Developmental stages | SRR13364372 | D-larva 2 |
| Developmental stages | SRR13364373 | Trochophore 5 |
| Developmental stages | SRR13364374 | Trochophore 4 |
| Developmental stages | SRR13364375 | Juvenile 10 |
| Developmental stages | SRR13364376 | Juvenile 8 |
| Developmental stages | SRR13364377 | Juvenile 7 |
| Developmental stages | RR13364378 | Juvenile 6 |
| Developmental stages | SRR13364379 | Pediveliger 16 |
| Developmental stages | SRR13364380 | Pediveliger 11 |
| Developmental stages | SRR13364381 | Pediveliger 10 |
| Developmental stages | SRR13364382 | Pediveliger 7 |
| Developmental stages | SRR13364383 | Umbo larva 9 |
| Developmental stages | SRR13364384 | Umbo larva 7 |
| Developmental stages | SRR13364385 | Trochophore 2 |
| Developmental stages | SRR13364386 | Trochophore 1 |
| Tissues | SRR10502224 | Labial palp |
| Tissues | SRR10502225 | Mantle |
| Tissues | SRR10502226 | Male gonad |
| Tissues | SRR10502227 | Haemocytes |
| Tissues | SRR10502228 | Gut |
| Tissues | SRR10502229 | Gill |
| Tissues | SRR10502230 | Foot |
| Tissues | SRR10502231 | Female gonad |
| Tissues | SRR10502239 | Digestive gland |
| Tissues | SRR10502240 | Adductor muscle |
| Antibiotics and bacteria | SRR13891783 | Gill-3, Exposure to a mixture of antibiotics (ampicillin, kanamycin, gentamycin, and streptomycin) for 24 h, 5 μg/L |
| Antibiotics and bacteria | SRR13891784 | Gill-2, Exposure to a mixture of antibiotics (ampicillin, kanamycin, gentamycin, and streptomycin) for 24 h, 5 μg/L |
| Antibiotics and bacteria | SRR13891785 | Gill-1, Exposure to a mixture of antibiotics (ampicillin, kanamycin, gentamycin, and streptomycin) for 24 h, 5 μg/L |
| Antibiotics and bacteria | SRR13891798 | Gill-3, *Vibrio parahemolyticus* challenge was performed 0.5 h after exposure to antibiotics for 24 h, 10^7^ CFU |
| Antibiotics and bacteria | SRR13891799 | Gill-2, *Vibrio parahemolyticus* challenge was performed 0.5 h after exposure to antibiotics for 24 h, 10^7^ CFU |
| Antibiotics and bacteria | SRR13891800 | Gill-1, *Vibrio parahemolyticus* challenge was performed 0.5 h after exposure to antibiotics for 24 h, 10^7^ CFU |
| Antibiotics and bacteria | SRR13891805 | Gill-3, without antibiotics or bacteria exposure |
| Antibiotics and bacteria | SRR13891816 | Gill-2, without antibiotics or bacteria exposure |
| Antibiotics and bacteria | SRR13891817 | Gill-1, without antibiotics or bacteria exposure |
| Antibiotics and bacteria | SRR13891811 | Gill-3, *Micrococcus luteus* challenge was performed 0.5 h after exposure to antibiotics for 24 h, 10^7^ CFU |
| Antibiotics and bacteria | SRR13891812 | Gill-2, *Micrococcus luteus* challenge was performed 0.5 h after exposure to antibiotics for 24 h, 10^7^ CFU |
| Antibiotics and bacteria | SRR13891813 | Gill-1, *Micrococcus luteus* challenge was performed 0.5 h after exposure to antibiotics for 24 h, 10^7^ CFU |
| Copper stress | SRR2895131 | Gills, exposure to copper sulfate, 0.2 ppm |
| Copper stress | SRR2895130 | Gills, without copper sulfate exposure |
| Ocean acidification stress | SRR9090063 | Mantles, sampled at time 40d, PH 8.1 |
| Ocean acidification stress | SRR9090064 | Mantles, sampled at time 40d, PH 7.4 |
